# A Modified Delphi consensus on early management of combined cardiovascular risk factors in adult patients with type 2 diabetes in Italy

**DOI:** 10.1007/s00592-026-02770-w

**Published:** 2026-08-10

**Authors:** Stefano Del Prato, Stefano Genovese, Carlotta Galeone, Paolo Mariani, Tommaso Acquaviva, Marco Antonutti, Massimiliano Anzaldi, Loredana Bucciarelli, Diego Carleo, Arturo Cesaro, Vera Frison, Enrico Gabellieri, Egidio Imbalzano, Marco Marini, Vincenzo Maria Monda, Nicola Riccardo Pugliese, Paolo Severino, Gian Pio Sorice, Mattia Squillace, Emanuele Tizzani, Alessia Ventricini, Pasquale Perrone Filardi

**Affiliations:** 1 Interdisciplinary Research Center ’Health Science’, Sant’Anna School of Advanced Studies, Pisa, Italy; 2 Centro Cardiologico Monzino IRCCS, Milan, Italy; 3 Bicocca Applied Statistics Center (B-ASC), University of Milano-Bicocca, Via Bicocca degli Arcimboldi 8, 20126 Milan, Italy; 4 Cardiac Surgery Unit, Department of Emergency and Organ Transplantation, University of Bari, Bari, Italy; 5 ASUFC (Azienda Sanitaria Universitaria Friuli Centrale) - SOC Cardiologia Ospedale di Udine Santa Maria della Misericordia, Udine, Italy; 6 Unit of Diabetology, Metabolic and Endocrine Diseases, “Cannizzaro” Emergency Hospital, Catania, Italy; 7 Pio Albergo Trivulzio, Milan, Italy; 8 International Center for T1D, Pediatric Clinical Research Center Romeo ed Enrica Invernizzi, Dipartimento di scienze Biomediche e Cliniche, Università di Milano, Milan, Italy; 9 Asl Napoli 2 Nord ds 43, Polo Diabetologico Casoria, Naples, Italy; 10 Department of Translational Medical Sciences, University of Campania “Luigi Vanvitelli”, Naples, Italy; 11 Division of Cardiology, A.O.R.N. “Sant’Anna e San Sebastiano”, Caserta, Italy; 12 Unit of Diabetology, Cittadella Hospital, ULSS6 Euganea, Cittadella, Italy; 13 Endocrine and Metabolic Diseases Unit, Santo Spirito Hospital, Casale Monferrato, Italy; 14 Department of Clinical and Experimental Medicine, University of Messina, Messina, Italy; 15 Cardiology and ICCU, AOU delle Marche, Ancona, Italy; 16 Diabetes Unit ’’Santissima Annunziata’’ Hospital Cento, Ferrara, Italy; 17 Department of Clinical and Experimental Medicine, University of Pisa, Pisa, Italy; 18 Department of Medical and Cardiovascular Sciences, Sapienza University of Rome, Rome, Italy; 19 Department of Precision and Regenerative Medicine and Ionian Area, Section of Internal Medicine, Endocrinology, Andrology and Metabolic Diseases, University of Bari Aldo Moro, Bari, Italy; 20 IRCCS Policlinico S. Donato, Milan, Italy; 21 Dipartimento di Cardiologia, Ospedale degli Infermi, Rivoli, Italy; 22 ASL Roma 2, Casa della Comunità di Via delle Averle, Roma, Italy; 23 Department of Advanced Biomedical Sciences, University of Naples Federico II, Naples, Italy

**Keywords:** Cardiovascular risk, Delphi consensus, Early treatment, Treatment adherence, Type 2 diabetes

## Abstract

**Background:**

Type 2 diabetes (T2D) is a complex chronic disease often associated with other metabolic conditions that increase the risk of cardiovascular (CV) disease and early mortality. Hence, early, integrated management of T2D may prevent long-term CV complications.

**Methods:**

To determine the appropriateness of early multifactorial intervention in people with T2D we conducted an online modified Delphi study. The study involved a multidisciplinary panel of 17 Italian experts, ensuring maintenance of study design and rigorous execution throughout the consensus process, in line with the core principles of Delphi studies. Main areas of investigation included: (1) targets of disease control for adults with T2D; (2) early treatment, CV risk assessment, and multi-factorial strategy; (3) unmet needs, including treatment adherence; and (4) public health aspects.

**Results:**

Consensus was unanimous (100% agreement) regarding T2D management based on the simultaneous control of glycemia, body weight, blood pressure, and lipids through an early multi-factorial approach as well as maintenance of target levels over time. Most panelists agreed on the need for a simplified therapeutic approach (88% agreement), supported by improved availability of appropriate pharmacological treatments and increased adherence (82% agreement).

**Conclusions:**

Italian experts strongly support the adoption of an early, multi-factorial approach to disease management to reduce long-term CV risk. To this end, the need for simplified treatment regimens and improved adherence to therapy emerged as key unmet needs.

## Introduction

Type 2 diabetes (T2D) is a complex chronic disease characterized by hyperglycemia that can lead to subsequent microvascular and macrovascular complications [1]. T2D is frequently associated with other metabolic conditions, particularly obesity, thereby increasing the risk of cardiovascular (CV) disease and early mortality [2, 3]. Accordingly, current guidelines on T2D management recommend individualized management strategies, rather than a rigid approach [4, 5]. Lifestyle and pharmacological interventions should be fitted to each patient, depending on the corresponding baseline CV risk, metabolic targets, and personal characteristics. The clinical benefits of controlling multiple modifiable metabolic factors, beyond hyperglycemia alone, are increasingly recognized [2, 6, 7]. Current guidelines recommend a holistic approach focused on organ protection [2, 6, 7], emphasizing the early use of cardio-renal protective treatments. These include sodium-glucose cotransporter 2 inhibitors (SGLT2i), glucagon-like peptide-1 receptor agonists (GLP-1 RAs), and newer dual glucose-dependent insulinotropic polypeptide (GIP)/GLP-1 receptor agonists. Such recommendations are based on the robust efficacy of these drug classes in reducing major adverse cardiovascular events and providing renal protection [4].

Despite growing evidence supporting the benefits of early intensive therapy on long-term morbidity and mortality [8–15] its clinical implementation - and, even more, the identification of optimal therapeutic target - remains debated. In this context, a rigorous, structured consensus methodology was adopted to generate expert agreement on clinical indications and implementation processes [16, 17].

## Materials and methods

We conducted a structured consensus study involving Italian experts, using a modified Delphi (e-Delphi) method aimed at identifying areas of strong agreements on T2D management, with focus on long-term CV risk [18].

The e-Delphi is a structured methodology that synthesizes expert perspectives in areas lacking robust quantitative evidence. The study adhered to established methodological standards for Delphi reporting (i.e., the ACCORD guidelines) and the core principles of Delphi studies [19, 20].

A preliminary non-systematic literature search (PubMed/MEDLINE, Google Scholar and grey literature) was conducted in June 2025 by one of the authors (C.G.) to identify gaps in knowledge and areas of uncertainty within the Italian context. Based on these findings, the following domains were defined: (1) Treatment targets in adults with T2D; (2) Early treatment, multifactorial strategies and CV risk; (3) Unmet needs, including treatment adherence; (4) Public health issues in Italy.

A Steering Committee (clinical experts: S.D.P., S.G., P.P.F.; methodological experts: C.G., P.M.) oversaw the study. The Committee met regularly via virtual meetings and email communication throughout all phases. Responsibilities included defining topics, developing questionnaires, establishing eligibility criteria, interpreting results, and drafting the manuscript. Committee members did not participate in voting.

The expert panel included cardiologists, diabetologists, endocrinologists, and internists. In order to ensure a real clinical practice expertise of participants and to limit the risk of selection bias, panelists were identified and invited on the basis of a priori defined, objective criteria. Eligibility criteria included: (1) clinical expertise, defined on the basis of participation of physicians in national and international multicentric real-world studies and clinical trials, in scientific articles published in peer-reviewed journals, in medical societies, and direct clinical experience with the management of T2D in their respective fields; (2) Different geographic distribution to ensure a broad representation across the country. No a priori target number of invitations was defined. Of 18 invited clinicians, 17 (94%) met the criteria and completed the study. Participation was voluntary, with no compensation provided.

A structured questionnaire was administered via SurveyMonkey^®^. Panellists were invited on November 12, 2025, and Round 1 was conducted from November 13–21, 2025. The questionnaire included multiple-choice items, and Likert-scale statements (‘strongly disagree’, ‘partially disagree’, ‘partially agree’, ‘strongly agree’). Agreement categories were collapsed for analysis. The aim of the questionnaire and the research objective were communicated in advance to all invited panelists via an introductory email and an explanatory page of the electronic questionnaire. The questionnaire aimed to explore experts’ opinions and clinical practices, assess consensus on pre-specified topics and examine the distribution of responses. All replies, collected during Round 1 and analyzed in an aggregated and anonymous form, reached full consensus during the first iteration; therefore, no second round was required, and the modified Delphi process was concluded.

Consensus criteria were established a priori. Consensus was defined as a level of agreement ≥ 66.7% of panelists. Descriptive statistical analyses were performed to summarize experts’ opinions, including frequency distributions and corresponding percentages for categorical variables, and mean or median values, standard deviations and ranges for continuous variables. Stratified analyses according to clinical specialization (cardiologists vs. non-cardiologists) were also performed.

This study did not involve the collection of sensitive data or patient participation. Therefore, according to Italian regulations, no ethical approval was required (Determine AIFA: 8th August 2024, https://www.gazzettaufficiale.it/eli/id/2024/08/20/24A04320/sg; 20th March 2008, https://www.gazzettaufficiale.it/eli/id/2008/03/31/08A02109/sg).

## Results

Out of a total of 17 participants, 8 (47.1%) were cardiologists, 6 (35.3%) were diabetologists and/or endocrinologists, 1 (5.9%) was an internist, 1 (5.9%) an internist/cardiologist and 1 (5.9%) an internist/diabetologist (Table 1). Participants were based in Northern (35.3%), Central (29.4%) and Southern (35.3%) Italy, and worked in university hospitals (29.4%), general hospitals (35.3%), scientific research institutes (IRCCS, 5.9%), or other settings such as local health units and private hospitals (29.4%). Most panelists worked in tertiary care settings (64.7%) and reported more than 10 years of experience managing people with T2D at high CV risk (76.5%). A median of 600 patients with T2D per panelist had been managed in the 12 months preceding participation in the Delphi study.

**Table 1:** Main characteristics of 17 participants to the Delphi consensus study

Characteristic	*n*	%
**Clinical specialization**		
Cardiologist	8	47.1%
Diabetologist/endocrinologist	6	35.3%
Internist	1	5.9%
Internist/cardiologist	1	5.9%
Internist/diabetologist	1	5.9%
**Geographic area**		
Northern Italy	6	35.3%
Central Italy	5	29.4%
Southern Italy/Isles	6	35.3%
**Institution/Hospital**		
University hospital	5	29.4%
General hospital	6	35.3%
Scientific research institute	1	5.9%
Other settings	5	29.4%
**Healthcare level**		
Primary care	1	5.9%
Secondary care	5	29.4%
Tertiary care	11	64.7%
**Years of clinical expertise in the field**		
< 5	1	5.9%
5–10	3	17.6%
> 10	13	76.5%

### Item 1—target values for CV risk factors in adult T2D subjects

82% of the panelists (*n* = 14) supported an HbA1c 6.0%−6.5% for reducing CV risk in people with T2D. Fifteen out of 17 experts (88%) agreed that HbA1c targets should be individualized based on patient and disease characteristics, particularly in the presence of established or concomitant CV disease (67% agreement).

A high level of agreement, if not full consensus, was achieved for clinical targets in CV prevention in T2D during the first Delphi round (Table 2). Fourteen out of 17 experts (82%) disagreed with the statement that HbA1c control is the sole therapeutic goal in adults with T2D, whereas 94% agreed that body weight reduction is a key component for CV prevention in overweight/obese T2D patients. Consensus (100%) was reached on the importance of blood pressure control (82% strongly agreed; 18% partially agreed), achievement of optimal lipid targets (94% strongly agreed; 6% partially agreed), and assessment of renal parameters (76% strongly agreed; 24% partially agreed) for CV risk management.

**Table 2:** Level of agreement on various statements focused on clinical targets for T2D management in relation to CV risk

Statement	Agree vs. disagree	% agreed/disagreed (total)	% strongly agreed/disagreed	% partially agreed/disagreed
Control of HbA1c is the only key goal in the management of adult patients with T2D	Disagree	82%	41%	41%
Body weight reduction is a relevant clinical target in the management of CV risk in adult patients with T2D who are:				
i) Slightly overweight (BMI: 25 - <27)	Agree	94%	24%	71%
ii) Highly overweight (BMI: 27 - <30)	Agree	94%	76%	18%
iii) Obese - Grade I (BMI: 30 - <35)	Agree	94%	82%	12%
iv) Obese - Grade II (BMI: 35 - <40)	Agree	94%	88%	6%
v) Obese - Grade III (BMI: ≥40)	Agree	94%	88%	6%
Blood pressure control should be part of an integrated prevention strategy of CV risk in adult patients with T2D	Agree	100%	82%	18%
Achieving optimal targets for plasma lipids (particularly LDL-C) is key in managing CV risk in adult patients with T2D	Agree	100%	94%	6%
Achieving optimal targets for kidney parameters (such as eGFR and ACR) is key in managing CV risk in adult patients with T2D	Agree	100%	76%	24%

Regarding kidney function, a high level of agreement (82%) was observed for estimated glomerular filtration rate (eGFR) and albumin-to-creatinine ratio (ACR), while 71% agreement was reached for metabolic dysfunction-associated steatotic liver disease (MASLD) as a target for CV risk reduction.

When we asked experts to provide some specific estimates related to their currently managed population of patients with T2D (based on subjective expert impression), it emerged that, on average: (i) 69% of their patients are estimated to have a BMI ≥ 27 kg/m^2^; (ii) 51% of their patients are estimated to achieve an HbA1c target below 7%; (iii) 45% of their patients are estimated to have atherosclerotic CV disease; (iv) 59% of their patients are estimated to achieve blood pressure targets, i.e., < 130 − 80 mmHg; (v) 57% of their patients are estimated to achieve LDL-C target, i.e., < 70 mg/dl. Further, according to 53% of panelists, simultaneous control of glycemia, body weight, blood pressure, and lipid profile is currently achieved in ≤ 30% of their managed population of patients with T2D. Simultaneous control of these parameters was less frequently reported by cardiologists (median: 15% of patients) than by other specialists (median: 37.5%).

### Item 2—early treatment, multi-factorial approach strategy and CV risk

**Fig. 1:**
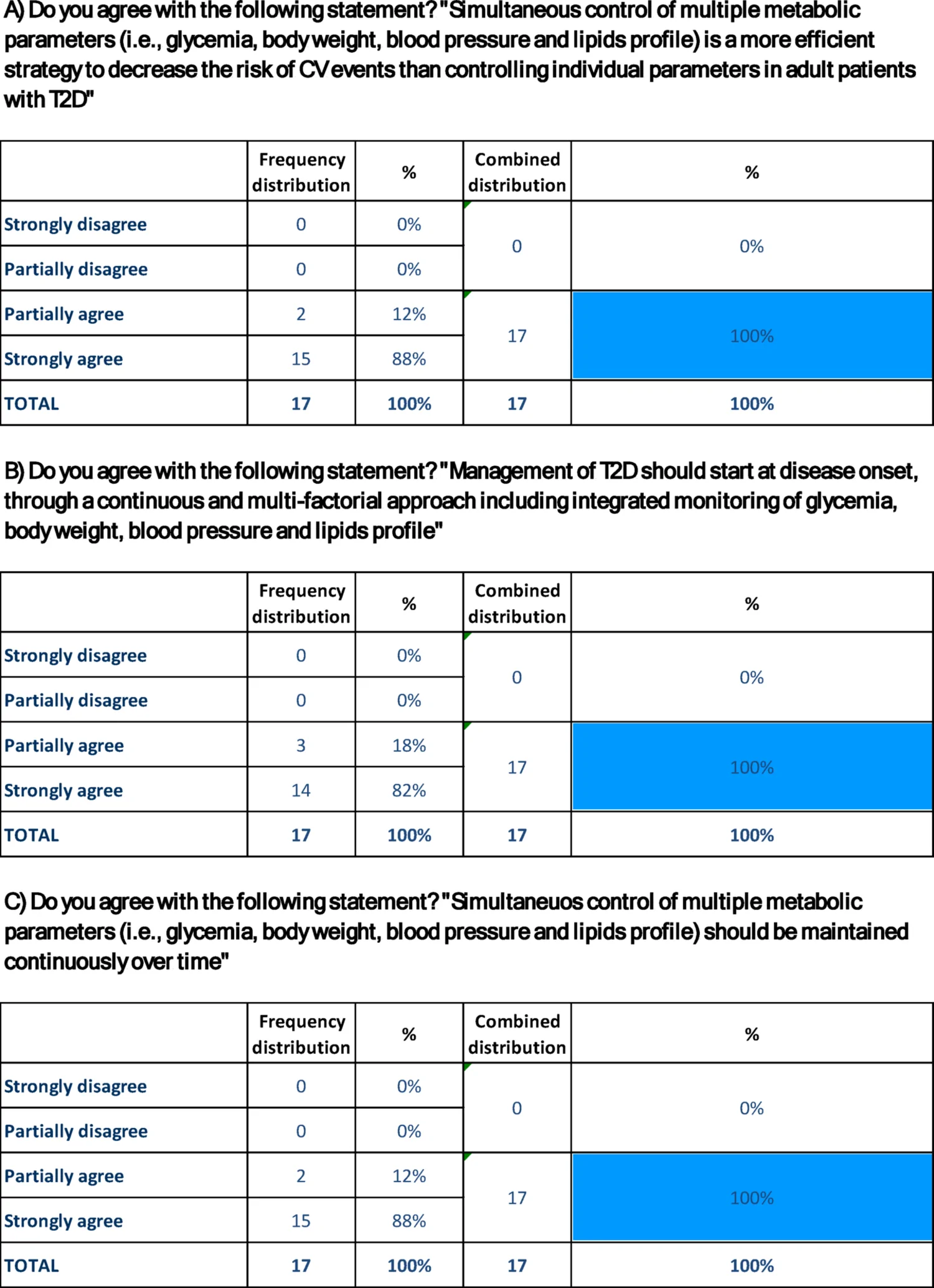
Level of agreement with selected statements on early treatment, multi-factorial approach strategy and CV risk. *CV* cardiovascular; *T2D* type 2 diabetes

Unanimous consensus (100%) supported the superiority of a multifactorial strategy compared with targeting a single CV risk factor (88% strongly agreed; 12% partially agreed). There was also strong agreement on the need of early implementation (82% strongly agreed; 18% partially agreed) and sustained maintenance (88% strongly agreed; 12% partially agreed) of target levels (Fig. 1). Maintaining multiple metabolic targets over time was considered a useful indicator of therapeutic effectiveness (76% strongly agreed).

Panelists agreed that the coexistence of two or more conditions (T2D, overweight/obesity, hypertension and dyslipidemia) may act synergistically and worsen cardiac function and structure, promoting cardiac remodeling (76% strongly agreed, 24% partially agreed).

Among the expected impact of early and effective treatment, panelists highlighted reduction in long-term risk of vascular complications (82% agreement) and mortality (71% agreement). Other outcomes — such as long-term remission (65%), improved adherence (65%), and weight reduction (59%) — did not reach the predefined consensus threshold.

### Item 3: treatment adherence and unmet needs

88% of panelists emphasized the need to simplify therapeutic strategies to improve adherence, particularly in patients with comorbidities and/or multiple metabolic abnormalities (87% agreement, Table 3). No consensus was reached on any specific age subgroup requiring more needs than others. 82% agreed on prioritizing the implementation of new treatments that can simplify therapeutic approach to improve therapeutic adherence.

**Table 3:** Level of agreement for questions on treatment adherence and unmet needs in adult patients with T2D

Question	Replies with ≥ 67% agreement	% agreement	Other frequent replies	% agreement
In your opinion, is there a clinical unmet need to simplify the therapeutic approach in the management of at least part of adult patients with T2D, also with the aim of improving treatment adherence?	Yes	88%	-	-
If yes, in which subgroup(s) of patients do you think there is such important unmet need?	1) Patients with comorbidities/with altered metabolic parameters	87%	2) Patients aged 75–84 years 3) Patients aged 45–64 years 4) Patients aged 65–74 years 5) Overweight/obese patients	53% 47% 40% 33%
In order to improve therapeutic adherence in adult patients with T2D, which aspect(s) do you consider a priority?	1) Availability of new treatments to simplify the therapeutic regimen	82%	2) Availability of treatments perceived as safe 3) Improving communication and the doctor-patient relationship	47% 47%
In your opinion, which are the main unmet needs in the management of adult patients with T2D at high/very high/extreme CV risk?	1) Improvements to achieve and maintain over time different metabolic targets together (i.e., glycemia, body weight, blood pressure, lipids)	71%	2) Increasing the adherence to lifestyle and monitoring recommendations 3) Promoting the integration of skills between different specialists (i.e., diabetologists, cardiologists, general practitioners)	59% 53%

Panelists also supported strategies facilitating simultaneous achievement and maintenance of metabolic targets (71% agreement). Lower levels of agreement were observed for improving adherence to lifestyle and monitoring recommendations (59%) and for multidisciplinary integration (53%).

### Item 4: public health aspects

**Fig. 2:**
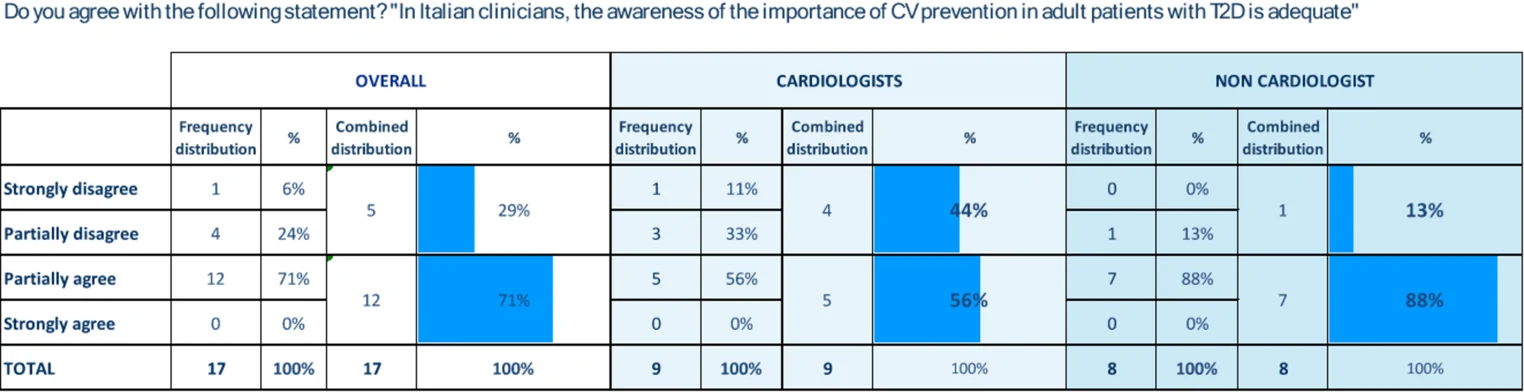
Level of agreement with a statement on the importance of CV prevention in patients with T2D, overall and according to the specialization of experts. *CV* cardiovascular; *T2D* type 2 diabetes

Twelve out of 17 experts (71%) partially agreed that awareness of the importance of CV prevention among Italian clinicians is adequate. Agreement was somewhat stronger among non-cardiologists than cardiologists (Fig. 2).

88% agreed that T2D management in Italy remains largely focused on glycemic control (glucocentric approach), often at the expense of comprehensive cardiovascular risk management.

Regarding multidisciplinary collaboration, agreement was highest for cardiologists (88%), diabetologists (82%), and general practitioners (76%), with lower agreement for nephrologists (59%).

## Discussion

This modified Delphi study highlights a strong consensus among multidisciplinary experts on the perceived need to adopt an early, multifactorial approach to T2D management, aimed at achieving sustained control of multiple metabolic parameters and reducing long-term CV risk. This high level of agreement is reflected by the findings that, for all statements related to early treatment, multifactorial strategies, and CV risk, the level of agreement was “strong” among more than three-fourths of experts. In addition to traditional CV risk factors, eGFR and ACR were also recognized by the panel as relevant markers to be addressed, reaffirming the clinical priority of comprehensive cardio-renal protection. The need for treatment simplification and improved adherence also emerged as a key priority, with a high level of agreement, among panelists. The consensus achieved by our panel is consistent with the current view that T2D is not merely a hyperglycemic condition, but rather a complex disease [1] requiring an integrated, holistic approach targeting a broad spectrum of CV risk factors, including obesity [6, 21–23]. The panelists highlighted the potential synergistic deleterious effects of uncontrolled metabolic abnormalities on cardiac function and structure. Although contemporary diabetes and cardiology guidelines recommend a comprehensive therapeutic approach, nearly 90% of panelists – without meaningful differences between specialists – agreed that, in Italy, clinical management remains predominantly focused on glycemic control. Although the latter remains essential, particularly for the prevention of microvascular complications, panelists unanimously recognized the value of an integrated care model for CV risk reduction.

Early intervention is crucial to counteract the so called “metabolic memory”, defined as the establishment of cellular processes due to initial poor metabolic control that limit the beneficial effects of subsequent improvements in the metabolic milieu achieved through treatment [12, 24]. Conversely, early intervention may confer a “legacy effect”, as demonstrated in landmark trials such as UKPDS [8] and STENO-2 trial [21]. Given that CV risk is largely driven by cumulative exposure to adverse metabolic factors, early intervention is widely considered the most effective strategy to reduce lifetime risk. Our panelists were strongly aligned with this concept, as reflected by the high degree of consensus regarding the importance of prompt implementation of a multifactorial approach and the need to extend as long as possible the maintenance of metabolic targets.

According to the ADA-EASD Consensus on the management of T2D [4], suboptimal adherence to glucose lowering therapy occurs in nearly half of the patients. In this context, the Delphi panel identified the availability of simplified therapeutic regimens as a key priority to improve adherence. Treatment simplification was recognized as an important clinical need, particularly in patients with comorbidities or complex metabolic profiles. The use of therapies capable of simultaneously targeting multiple metabolic abnormalities may help to address this need. For example, SGLT2i offer a multi-targeted approach by improving glycemic control, reducing blood pressure and promoting weight loss, which provide direct cardiorenal protection, including an impact on the risk of hospitalization for heart failure. Similarly, GLP1-RA and newer dual GLP1-RA/GIP simultaneously improve glycemic control, body weight, and systemic vascular factors such as blood pressure and lipid profile, thereby conferring independent CV benefits [4, 25–27].

Panelists also emphasized the difficulty of achieving comprehensive metabolic control in clinical practice. According to more than half of respondents, on the basis of subjective impressions about their currently managed set of patients with T2D, simultaneous control of all major metabolic parameters is achieved in ≤ 30% of patients, and approximately half of patients do not reach an HbA1c target < 7%. These findings underscore the need for more effective and integrated therapeutic strategies. The management of T2D has been evolving alongside advancements in available treatment options and future promising therapies aimed at comprehensive metabolic and organ protection.

This study also explored the Italian clinical context. Overall, there was general agreement regarding the perceived awareness of the importance of CV prevention in people with T2D within the Italian medical community; however, noticeable differences emerged between specialists, with 44% of cardiologists expressing disagreement with this statement (i.e., they disagreed that the current level of awareness is adequate in Italy). This divergence in perception highlights the need for targeted educational initiatives to bridge this gap.

The consensus achieved on early, multifactorial treatment using newer pharmacological agents raises questions regarding pharmacoeconomic sustainability and treatment accessibility within the Italian healthcare system. While the upfront costs of such therapies are higher compared to traditional drugs, and may impact economic expenses in the short-term, their implementation must also take into account the potential economic benefits deriving from the prevention of cardio-renal complications and hospitalizations in the long-term [28, 29]. With further reference to treatment accessibility, the decentralization of the Italian healthcare system across different Regions may introduce logistical and administrative challenges, thus complicating a consistent access to these therapies throughout the Country.

This study shares the inherent limitations of consensus-based methodologies [16]. Specifically, the findings are based on expert opinion rather than primary clinical data and therefore represent a lower level of evidence. In addition, all participants were based in Italy: although the topics addressed are broadly applicable, the results may reflect national clinical perspectives and may not be fully generalizable internationally.

In conclusion, while reflecting established clinical evidence, this Delphi consensus study highlights a strong agreement among Italian experts regarding the importance of early, multifactorial management of T2D and draw attention to key unmet needs, including treatment simplification and improved adherence. These insights may contribute to increasing awareness of CV risk and promoting a more integrated therapeutic approach focused on comprehensive cardio-renal protection within the Italian clinical community.
